# Supplementary material for: CRISPRing KRAS: A Winding Road with a Bright Future in Basic and Translational Cancer Research
Source: Cancers (Basel). 2024 Jan 22;16(2):460. doi: 10.3390/cancers16020460 (PMC10814442; doi:10.3390/cancers16020460)
Supplement: Supplementary file 1 [file cancers-16-00460-s001.zip › cancers-2830417-supplementary.pdf]

**Table S1.** KRAS mouse modeling by the CRISPR/Cas system (in chronological order).

| Tumor type | Targeted gene | Delivery | Methods | Publication year | Reference |
|---|---|---|---|---|---|
| Lung adenocarcinoma | Rosa26, Myd88, A20, $Kras^{G12D}$, p53, LKB1 | AAV | Intranasal/intratracheal | 2014 | [96] |
| Pancreatic cancer | LKB1 | Lentivirus | Retrograde ductal injections | 2015 | [97] |
| Pancreatic cancer | Brac2 | Electroporation | Laparotomy | 2016 | [25] |
| Colorectal cancer | Apc, Trp53 | Lentivirus | Mucosal injection | 2017 | [98] |
| Gallbladder cancer | Cr8, p53, PTEN | Retroviral | Subcutaneous /orthotopic transplantation of organoids | 2019 | [99] |
| Pancreatic cancer | Kras, Trp53, LKB1, Arid1a | AAV | Surgical procedure and direct injection | 2019 | [100] |
| Colorectal cancer/pancreatic cancer/ | $Kras^{G12R}$, $Kras^{G13D}$, $Kras^{G12C}$ | Nucleofection | Blastocyst injection | 2020 | [101] |
| Gall bladder cancer | $Kras^{G12D}$, Trp53 | Lentivirus | Implantation of organoid-derived tumor bud | 2021 | [102] |
| Pancreatic cancer | Kras | Lentivirus | Orthotopic injection | 2021 | [103] |
| Non–small cell lung cancer | Trp53, $Kras^{G12D}$ | AAV | Intratracheal | 2021 | [104] |
| Mammary tumor | $Kras^{G12D}$, Pik3ca | AAV | Intraductally into mammary glands | 2023 | [105] |
| Lung cancer /pancreatic cancer | $Rosa26^{PE2}$, Trp53 | Lentivirus | Intratracheally transduced/retrograde pancreatic duct viral delivery | 2023 | [106] |

AAV, Adeno-associated virus;
